# Active elastocapillarity in soft solids with negative surface tension

**DOI:** 10.1126/sciadv.abk3079

**Published:** 2022-03-11

**Authors:** Jack Binysh, Thomas R. Wilks, Anton Souslov

**Affiliations:** 1 Department of Physics, University of Bath, Claverton Down, Bath BA2 7AY, UK.; 2 School of Chemistry, University of Birmingham, Edgbaston, Birmingham B15 2TT, UK.; 3 Exact Sciences Innovation, Sherard Building, Edmund Halley Road, Oxford OX4 4DQ, UK

## Abstract

Active solids consume energy to allow for actuation, shape change, and wave propagation not possible in equilibrium. Whereas active interfaces have been realized across many experimental systems, control of three-dimensional (3D) bulk materials remains a challenge. Here, we develop continuum theory and microscopic simulations that describe a 3D soft solid whose boundary experiences active surface stresses. The competition between active boundary and elastic bulk yields a broad range of previously unexplored phenomena, which are demonstrations of so-called active elastocapillarity. In contrast to thin shells and vesicles, we discover that bulk 3D elasticity controls snap-through transitions between different anisotropic shapes. These transitions meet at a critical point, allowing a universal classification via Landau theory. In addition, the active surface modifies elastic wave propagation to allow zero, or even negative, group velocities. These phenomena offer robust principles for programming shape change and functionality into active solids, from robotic metamaterials down to shape-shifting nanoparticles.

## Teaser

Active surface stresses program shape and function into 3D elastic solids.

## INTRODUCTION

Embedding stress-generating active elements into a passive solid powers functionality inaccessible in thermal equilibrium. These active metamaterials occupy the space between materials and machines, giving rise to exotic phenomena from actuation and shape change (*1*–*13*) to overdamped wave propagation (*14*–*17*) and nonreciprocal interactions (*18*–*20*). For two-dimensional (2D) active interfaces, powerful design principles exist for the distribution and control of stress-generating elements to achieve a target behavior (*11*, *12*, *21*–*24*). A key challenge is to develop these principles for the control of bulk 3D solids. Realizations of far-from-equilibrium solids range from macroscopic mechanical materials (*9*, *18*) and hydrogels (*25*–*27*) down to the microscale (*3*, *5*, *23*, *13*). The challenge of spanning these systems and length scales requires principles based on a continuum approach.

In thermal equilibrium, the shape and structure of a soft solid is determined not only by 3D bulk elasticity but also by surface stresses on its 2D boundary. This competition, termed (passive) elastocapillarity (*28*, *29*), has been used to stiffen composites (*30*), self-assemble micro-objects (*31*, *32*), and drive the coiling of nanoparticle helices (*33*). These phenomena all originate in the minimization of surface area due to the isotropic surface stress tensor ${ϒ}_{\mathrm{ij}}^{p}=\gamma_{p}\delta_{\mathrm{ij}}$, where δ*_ij_* is the Kronecker delta. Because passive elastocapillary solids are in equilibrium, their surface tension γ*_p_* is constrained to be positive.

**Fig. 1.**
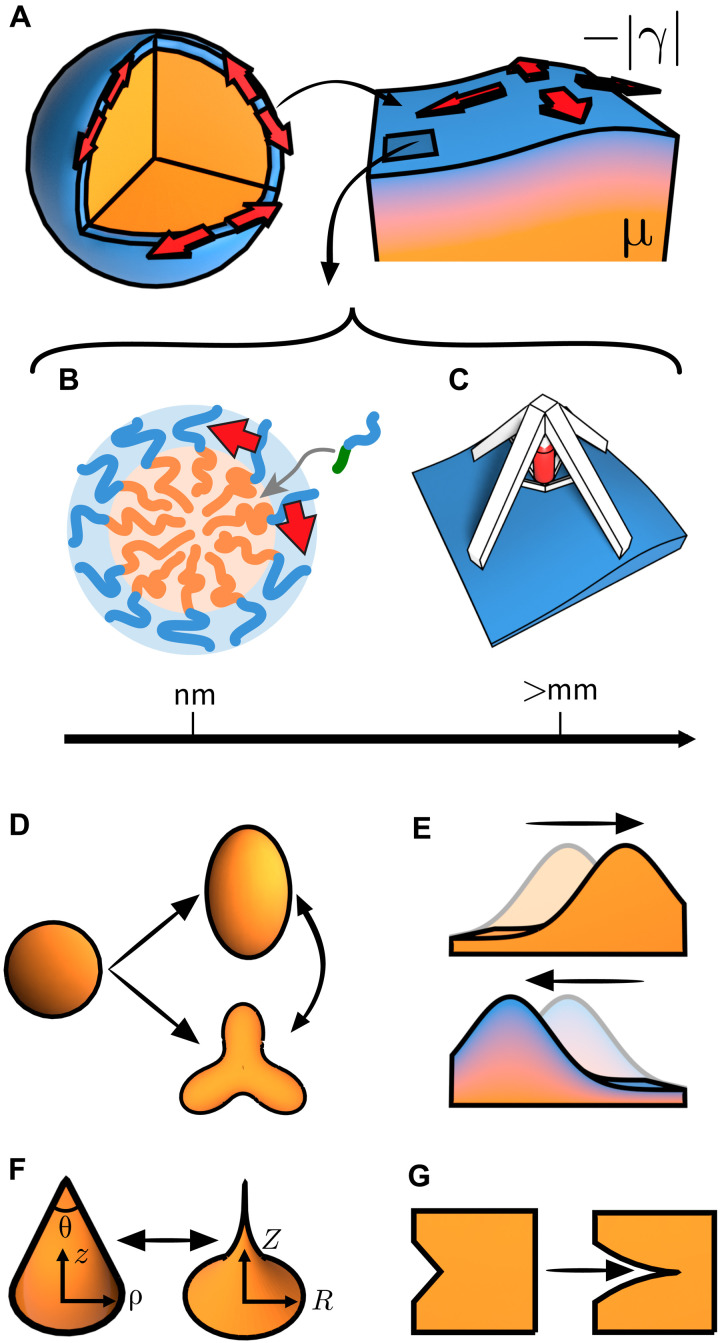
Active elastocapillary phenomena in soft solids with negative surface tension. (**A**) At the boundary of a bulk 3D soft solid, an active layer (blue) exerts dilational surface stresses (red arrows), which compete against bulk elasticity (orange). Left: A cutaway view of the entire solid. Right: A zoom in near the boundary. (**B** and **C**) Mechanisms leading to active surface stresses, from the nanoscale to the macroscale. (B) Insertion of complementary polymers into a nanoparticle surface causes steric crowding (*13*). (C) An active metamaterial in which mechanical actuators are embedded in the surface of an elastic medium (*11*, *12*, *18*). (**D** to **G**) Continuum phenomenology. (D) Switching between spheres and complex shapes of higher surface area. (E) Negative group velocity of elastocapillary Rayleigh waves. (F) Sharpening of corners and edges, with a cone of tip angle θ deforming into a cusp of the form ∣*Z*(*R*)∣∼ θ^−1^*R*^1/4^. (G) Promotion of crack propagation.

In this work, we show that elastocapillarity offers a distinct set of design principles when considering 3D soft solids driven out of equilibrium. The interplay between bulk and surface stresses translates to this far-from-equilibrium context, but now, each stress tensor may acquire additional, active terms (*14*, *22*). We refer to the resulting phenomenology as active elastocapillarity. This scenario stands in contrast to the buckling of elastic shells (*4*, *34*–*37*) or morphing of thin programmable sheets (*23*, *24*, *38*, *39*). Rather than a thin layer, we consider a fully 3D solid (Fig. 1A), which is sufficiently soft for active surface stresses to strain the entire bulk. We focus on a minimal change to the surface stress tensor due to activity. This is the addition of an isotropic dilational stress ${ϒ}_{\mathrm{ij}}^{a}\equiv \gamma_{a}\delta_{\mathrm{ij}}$, where γ*_a_* < 0. Sufficiently strong dilations will overpower passive tension γ*_p_*, giving an overall surface tension γ, which is effectively negative, γ ≡ γ_a_ + γ_p_ < 0 (*37*, *40*, *41*).

Intuitively, positive surface tension rounds any 3D object into a sphere. For negative surface tension, does a unique favored shape exist? Developing a continuum theory of active elastocapillarity, here, we show that final shape is not determined by∣γ∣alone. Instead, the shape can be selected by varying either surface stresses or bulk elastic moduli. As active driving increases, we find that distinct geometries discontinuously snap between one another, as realized in a simple particle-based numerical model. As well as shape, active surface stresses also control dynamic phenomena. For example, we find that negative surface tension softens Rayleigh wave propagation, leading to zero (or even negative) group velocity. Together, our results form a toolkit for programming the functionality of 3D active solids.

### Experimental realizations

In Fig. 1 (B and C), we give examples of experimental mechanisms for how active surface stresses can be designed. At the microscale, Fig. 1B shows a nanoparticle made of a diblock copolymer with a hydrophilic head (blue) and a hydrophobic tail (orange). The long tails form a melt, leading to bulk elasticity inside the nanoparticle (*13*). Insertion of a complementary polymer (blue head and green tail) into the nanoparticle surface drives area growth. As an example, Hua *et al.* (*13*) achieved this insertion using binding between paired DNA nucleobases (orange to green). To accommodate this growth, the nanoparticles deform away from their equilibrium spherical shape.

On the macroscale, a simpler realization is to embed dilational motors into the surface of a solid rubber ball (or another object), as shown in Fig. 1C. Alternatively, macroscale surface dilation can be designed using self-folding origami sheets or other deployable structures that spontaneously grow their effective surface area as they unfold (*11*, *12*). The incompressibility of the underlying rubber ball prohibits isotropic expansion at fixed spherical shape. Instead the ball shears, with the coupling between rubber shear modulus and dilational surface stresses in the active sheet determining the final shape of the composite object.

We focus on physical realizations using active elements such as independent motors. Similar to all examples of active matter, these motors locally consume energy to exert mechanical stresses. When these decentralized stresses couple together, shape change can become an emergent phenomenon via spontaneous symmetry breaking. An alternative mechanism for generating negative surface tension commonly used to make wrinkling patterns (*25*, *26*, *37*, *42*–*49*) is to globally prestress a planar sheet, which is then glued onto a flat substrate (*50*). By contrast, active elements can lead to a negative surface tension even when embedded into the surface of an arbitrarily shaped object. These dilational elements are readily found across many length scales (*12*, *13*). Here, we take a continuum approach that is independent of the microscopic origin of negative surface tension or system scale. For example, in Fig. 1B, control over the continuum-level surface tension γ can be implemented by varying the concentration of inserted polymer. In Fig. 1C, such control can be achieved by varying the forces exerted by the macroscopic motors.

## RESULTS

Figure 1 (D to G) illustrates a selection of the active elastocapillary phenomena that arise because of spontaneous growth of surface area. Below, we focus on exact solutions for deformations of a sphere (Fig. 1D) and surface waves and instabilities (Fig. 1E). First, we give an example that typifies the phenomenology of active elastocapillarity: the sharpening of a cone to a cusp (Fig. 1F).

Passive elastocapillarity smooths out a vertical cone of tip angle θ (*51*–*55*). By contrast, active elastocapillarity will sharpen this feature to a power law cusp. The solid’s boundary is then described by the curve ∣*Z*(*R*)∣∼θ^−1^*R*^1/4^, with the deformed conical height *Z* converging sharply to the origin as a function of its deformed radius *R*. To derive this power law, we consider the elongation of an incompressible elastic cylinder, of elastic modulus μ and undeformed radius ρ, under the action of negative surface tension on its curved boundary. The stretch factor λ > 1 is found by balancing the elastic deformation energy μρ^2^λ^2^ against the surface energy $|\gamma |\rho \sqrt{\lambda }$ to give λ ∼ (∣γ∣/μρ)^2/3^. The deformed height is then *Z* = λ*z*, with a corresponding radial contraction of $R=\rho /\sqrt{\lambda }$. We then take the cone to be a stack of infinitesimally thin cylinders of progressively decreasing radius ρ ∼ θ*z*. Each cylinder experiences a *z*-dependent elongation λ(*z*) ∼ (θ*z*)^−2/3^. Integrating these extensions and expressing the result in terms of the deformed radius, *R* ∼ θ*z*λ(*z*)^−1/2^ ∼ (θ*z*)^4/3^, yields the *R*^1/4^ power law. In the Supplementary Materials, we describe this theoretical approach in detail and provide in fig. S1 a numerical demonstration of the cone-to-cusp transition, as well as confirmation of the *R*^1/4^ power law. Sharpening of corners corresponds to the case of a small conical angle θ. Taking a large θ instead corresponds to crack proliferation through stress concentration, shown in Fig. 1G.

### Continuum theory

We now proceed to the general framework of our continuum description. Active elastocapillarity is defined by two intrinsic length scales. The first, so-called elastocapillary length *l*_γ_ ≡ ∣γ∣/μ, measures the ratio of effective surface tension ∣γ∣ to shear elastic modulus μ of the solid. Intuitively, at length scales larger than the elastocapillary length *l*_γ_, 3D elasticity stabilizes the surface. What happens at ever smaller scales? In passive elastocapillarity, stability is provided by positive surface tension. By contrast, within active elastocapillarity, the destabilizing contribution of negative surface tension γ must be regularized by higher-gradient stresses. We focus here on surface effects and introduce a second bendoelastic length *l*_κ_ ≡ (κ/μ)^1/3^. This length scale can arise, for example, from a surface with bending modulus κ (*56*) or from the length dependence of active stresses γ_a_. A distinct stabilization mechanism is bulk dispersion. In the Supplementary Materials, we consider the effects of a shear modulus μ(*q*) = μ_0_ + μ_1_*q*^2^, dependent now on the planar wave number *q*, which instead leads to a stabilizing length scale $\sqrt{\mu_{1}/\mu_{0}}$. We contrast this effect with viscous dissipation, from which no such stabilization is possible.

**Fig. 2.**
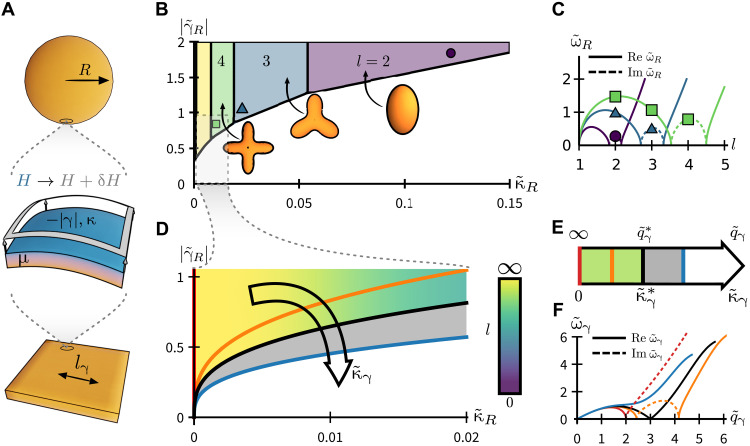
Shape instability and wave propagation within active elastocapillarity. (**A**) Schematic of an elastic solid with shear modulus μ and surface bending rigidity κ deformed by a negative surface tension −∣γ∣, which gives excess mean curvature *H* → *H* + δ*H*. (**B**) Phase diagram for the stability of an active elastocapillary sphere of radius *R*, controlled by rescaled surface tension $|{\tilde{\gamma }}_{R}|[\equiv |\gamma |/(\mu R),\text{corresponding to activity}]$ and bending modulus ${\tilde{\kappa }}_{R}$ [≡κ/(μ*R*^3^)]. The border between the white and the colored regions corresponds to activity at which the sphere goes unstable. Color indicates the dominant azimuthal mode number *l*, with insets showing unstable modes at *l* = 2,3, and 4. (**C**) Dispersion ${\tilde{\omega }}_{R}\equiv \omega R\sqrt{\rho /\mu }$ of spherical oscillations corresponding to points marked by square, triangle, and circle in (B). (**D** and **E**) The limit *l* → ∞ describes a half-space, with 1D phase diagram (E) controlled by rescaled bending modulus ${\tilde{\kappa }}_{\gamma }\equiv \kappa \mu^{2}/{|\gamma |}^{3}$. Colored curves (red, orange, black, and blue) and regions (green and gray) of (D) map to corresponding points and regions of (E). At ${\tilde{\kappa }}_{\gamma }^{*}=1/27$, the half-space destabilizes at rescaled wave number ${\tilde{q}}^{*}=3$ [(E), green region]. For ${\tilde{\kappa }}_{\gamma }≲1.5\;{\tilde{\kappa }}_{\gamma }^{*}$, ${\tilde{q}}_{\gamma }≳0.8\;{\tilde{q}}_{\gamma }^{*}$, we find negative group velocity surface waves [(E), gray region]. (**F**) Surface wave dispersions ${\tilde{\omega }}_{\gamma }\equiv \omega \sqrt{\rho \gamma^{2}/\mu^{3}}$ corresponding to colored lines in (D) and (E).

An object’s shape results from the competition between bulk elasticity and boundary conditions containing active surface stresses. We solve the equations of linear elastodynamics with a stress-matching condition at the surface (*57*–*59*)

$$-\sigma_{\mathrm{nn}}=-2(\gamma -\kappa \nabla^{2})\delta H$$ (1)

for slow variations in initial curvature *H*, where σ*_nn_* is the component of the 3D elastic stress tensor normal to the surface, ∇^2^ is the covariant (surface) Laplacian, and δ*H* is the variation of mean curvature (see Fig. 2A). In “Linear instability and shape change in an active elastocapillary droplet” section, we discuss the derivation of Eq. 1 and details of our solutions. Notably, our approach accounts for 3D elastic coupling between active surface elements. This allows us to describe deformations that are inaccessible when treating bulk elasticity as an external 2D potential coupled to a surface energy [cf. (*41*, *46*–*49*, *60*–*63*)]. We contrast these approaches further in the Supplementary Materials.

For any shape of size *R*, the solutions that we find must be characterized by the length scale triplet (*l*_γ_, *l*_κ_, *R*). We then define two independent dimensionless ratios as the surface tension and bending modulus rescaled by the size *R*: ${\tilde{\gamma }}_{R}\equiv \gamma /(\mu R)=\text{sign}(\gamma )l_{\gamma }/R$ and ${\tilde{\kappa }}_{R}\equiv \kappa /(\mu R^{3})={\left(l_{\kappa }/R\right)}^{3}$. When size *R* is too large to be relevant (such as for an infinite half-space), the solutions depend only on the quantity ${\tilde{\kappa }}_{\gamma }=\kappa \mu^{2}/{|\gamma |}^{3}={\left(l_{\kappa }/l_{\gamma }\right)}^{3}$. Here, ${\tilde{\kappa }}_{\gamma }$ describes the ratio of stabilizing elasticity (μ and κ) to destabilizing activity ∣γ∣. We conclude that under overall rescaling, the phenomenology remains unchanged and that continuum elastocapillarity remains valid across any scale, from nanoparticles to macroscopic metamaterials.

### Tunable instabilities and shape selection

Spheres minimize area at fixed volume, and positive surface tension drives every initial shape toward that of a sphere. By contrast, negative surface tension drives transitions away from a sphere into a variety of shapes. Our exact results demonstrate how to select between these shapes using the elasticity of the underlying solid. The phase diagram in Fig. 2B shows that for low active driving, $|{\tilde{\gamma }}_{R}|$ is small and spherical shapes are stable. As activity increases, spheres spontaneously destabilize. The threshold activity for instability, encoded in $|{\tilde{\gamma }}_{R}|$, and the azimuthal wave number *l* of the dominant unstable mode are both determined by the balance of surface and 3D moduli ${\tilde{\kappa }}_{R}$ (∼κ/μ).

When the bending modulus is small compared to 3D elasticity (small ${\tilde{\kappa }}_{R}$), we find an instability at wavelength ∼*l*_κ_, allowing for control over fine structure and surface texture. The threshold $|{\tilde{\gamma }}_{R}|$ and wave number of this instability map to those of the wrinkling instability of a stiff, prestressed layer bonded onto a substrate (*27*, *37*, *42*, *44*–*48*, *50*). To make this mapping, we identify the bendoelastic length *l*_κ_ with the physical thickness of the stiff layer and the negative surface tension γ with a uniform dilational stress within the layer, as described in the Supplementary Materials.

However, as bulk elasticity weakens (large ${\tilde{\kappa }}_{R}$), both the wavelength and penetration depth of these wrinkles become comparable to the system size. In this limit, we cannot describe the instability as that of a nearly planar surface, and our approach via fully 3D elasticity becomes essential (see the Supplementary Materials). Here, we find destabilization at the level of global shape change (Fig. 2B, right-hand side). In particular, at the largest ${\tilde{\kappa }}_{R}$, we find shapes with uniaxial anisotropy, which are inaccessible, for example, via instabilities at fixed surface area (*43*). These unstable modes originate in the dispersion relations ${\tilde{\omega }}_{R}(l)$ shown in Fig. 2C; here, *l* is the azimuthal wave number, and frequency ${\tilde{\omega }}_{R}\equiv \omega R\sqrt{\rho /\mu }$ is rescaled by the sphere radius *R* and transverse speed of sound $c_{T}=\sqrt{\mu /\rho }$ (with ρ as the bulk density). Figure 2C shows dispersions corresponding to points marked by square, triangle, and circle in Fig. 2B, just above the onset of instability. In each case, as threshold negative surface tension ${\tilde{\gamma }}_{R}$ is crossed, a single mode is driven unstable ($\text{Im}\;{\tilde{\omega }}_{R}>0$, dashed line). This instability gives a scale-free tool for designing global shape change in bulk 3D solids, distinct from the buckling of thin elastic shells (*35*).

For a flat object, energy injection will soften surface waves and drive wrinkling instabilities [see Fig. 2 (D and E)]. Figure 2D shows a zoom-in of the *l* → ∞ (equivalently *R* → ∞) limit of Fig. 2B. In this limit, the phase diagram collapses to the 1D diagram shown in Fig. 2E, controlled solely by the rescaled bending modulus ${\tilde{\kappa }}_{\gamma }\equiv \kappa \mu^{2}/{|\gamma |}^{3}$: Each curve ${\tilde{\gamma }}_{R}∼{\tilde{\kappa }}_{R}^{1/3}$ in Fig. 2D maps to a value of ${\tilde{\kappa }}_{\gamma }$ in Fig. 2E (colored bars). At a threshold ${\tilde{\kappa }}_{\gamma }^{*}$, the half-space destabilizes (Fig. 2E, green region), which, in unscaled variables, occurs when active driving ∣γ*∣ = 3(κμ^2^)^1/3^. The length scale for this instability is characterized by a rescaled planar wave number ${\tilde{q}}_{\gamma }\equiv q|\gamma |/\mu$ (Fig. 2E). At instability, the critical wave number ${\tilde{q}}_{\gamma }^{*}=3$ or in unscaled units $q^{*}=l_{\kappa }^{-1}$.

Even below instability, a vestige of surface activity can be measured via the negative group velocity of surface elastocapillary waves (Fig. 2E, gray region). In Fig. 2F, we show the planar dispersion relation ${\tilde{\omega }}_{\gamma }({\tilde{q}}_{\gamma })$ in which the frequency ω is now rescaled by both the elastocapillary length *l*_γ_ and the speed of sound *c_T_*. For weak active driving (large ${\tilde{\kappa }}_{\gamma }$), the half-space is stable and plane waves stiffen at high wave numbers. As active driving increases (${\tilde{\kappa }}_{\gamma }$ decreases), energy injection softens high wave numbers, leading first to negative group velocity $d{\tilde{\omega }}_{\gamma }/d{\tilde{q}}_{\gamma }<0$ and then to full-blown surface instability. Intuitively, this behavior stems from an effective shift of the shear modulus by negative surface tension, μ → μ − ∣γ∣ *q* (see the “Waves and instabilities in an active elastocapillary half-space” section). This rescaling causes μ, restoring elastic forces, and phase velocities all to vanish on a scale set by the elastocapillary length *l*_γ_.

The instability thresholds for wave number q* and active driving ∣γ*∣ can both be tuned using the surface modulus κ and 3D shear modulus μ. In other words, by selecting the material parameters of the passive solid, we can select the first mode that goes unstable once activity is turned on.

### Nonlinear elasticity and universality

Linear analysis can select only mode number, not mode amplitude. This amplitude is typically selected by nonlinear terms, which break symmetry. For example, geometric curvature of a surface will break the up-down symmetry of normal displacements, leading to the selection of dimples or labyrinthine structure in wrinkling (*46*–*48*). Here, we explore a distinct symmetry breaking mechanism: bulk material nonlinearity. Nonlinear elastic response will break the symmetry between positive and negative amplitude deformations present in our linear analysis. Thereby, nonlinearity selects global shape.

**Fig. 3.**
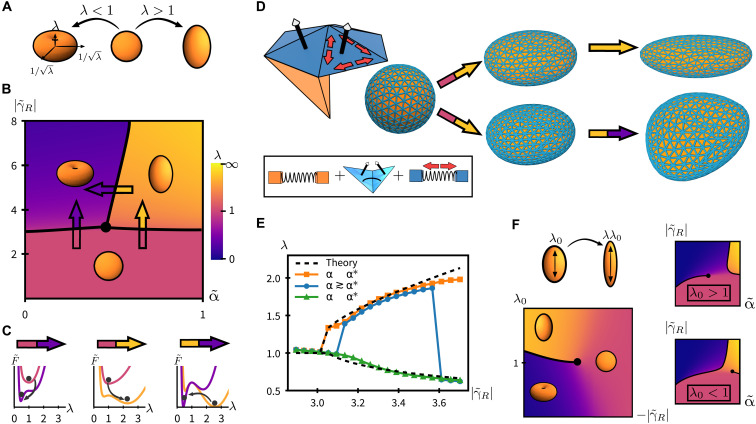
Nonlinear active elastocapillarity selects mode amplitude. (**A**) Homogeneous deformations are described by stretch factor λ, with λ > 1 giving a worm and λ < 1 giving a pancake. (**B**) Worm/pancake phase diagram, in activity $|{\tilde{\gamma }}_{R}|$ and material nonlinearity $\tilde{\alpha }$. Spheres destabilize as $|{\tilde{\gamma }}_{R}|$ increases, but the resulting shape (worm/pancake) depends on $\tilde{\alpha }$. At fixed $\tilde{\alpha }$, worms suffer a second snap-through transition at large ${\tilde{\gamma }}_{R}$. These discontinuous transitions meet at a critical point. (**C**) Landscapes for effective energy describing discontinuous transitions along the arrows marked in (B). (**D** and **E**) Simulations of minimal ball-spring model demonstrate continuum theory predictions (see movie S1). By tuning spring-level nonlinearity, we realize worms (top), pancakes, and snap-through (bottom). (**E**) Theory-simulation comparison for dependence of λ on active driving $|{\tilde{\gamma }}_{R}|$. For numerical data, orange squares and blue triangles correspond to data shown in (**D**). Theoretical fit shows all (meta)-stable minima for the Mooney-Rivlin continuum theory, Eq. 2, with parameters matched to the orange squares ($\tilde{\alpha }>{\tilde{\alpha }}^{*}$). (**F**) An anisotropic shape, with initial stretch factor λ_0_, splits the critical point from part (B). For both worm (λ_0_ > 1, top right) and pancake (λ_0_ < 1, bottom right), initial anisotropy either grows smoothly or snaps through, depending on the path in parameter space. A cut in $|{\tilde{\gamma }}_{R}|-\lambda_{0}$ space through the critical point (bottom left) gives an Ising-like transition described by Landau theory, Eq. 3.

We focus on the *l* = 2 mode of uniaxial deformations. The amplitude of this uniaxial strain can be approximated by a homogeneous deformation with principal stretch factor λ (see Fig. 3A). Stretch factor λ > 1 corresponds to a worm-like (prolate) shape, and λ < 1 corresponds to a pancake-like (oblate) one. A general nonlinear elasticity introduces an infinite set of materials parameters, making inaccessible an exact solution such as the one that we obtained in the linear regime. In keeping with the minimal approach taken thus far, we first consider a simple model of nonlinear elasticity that accounts for the effects of material nonlinearity: the Mooney-Rivlin model, often used for rubbers and polymer gels such as those shown in Fig. 1 (B and C) (*64*). We will return to the importance of this choice below. An effective energy $\tilde{F}\equiv F/\mu R^{3}$ for this far-from-equilibrium solid is given by

$$\tilde{F}={\tilde{F}}_{\text{NH}}+{\tilde{F}}_{\text{MR}}+{\tilde{F}}_{\text{bend}}+{\tilde{\gamma }}_{R}\tilde{A}$$ (2)

For the active surface contribution ${\tilde{\gamma }}_{R}\tilde{A}$, we take the area $\tilde{A}$ of a uniaxial ellipsoid (Fig. 3A). This is balanced by a Helfrich bending energy ${\tilde{F}}_{\text{bend}}$ (*65*) and equilibrium bulk elasticity, composed of the neo-Hookean contribution ${\tilde{F}}_{\text{NH}}\equiv (1-\tilde{\alpha })(2\lambda^{-1}+\lambda^{2})/2$ and the Mooney-Rivlin term ${\tilde{F}}_{\text{MR}}\equiv \tilde{\alpha }(\lambda^{-2}+2\lambda )/2$. We give expressions for the surface and bending energies in terms of λ in the “Nonlinear theory” section. Crucially, this model includes a single parameter $\tilde{\alpha }$ to continuously tune material nonlinearity, which ranges between the neo-Hookean limit of only geometric nonlinearity, $\tilde{\alpha }=0$, and maximal nonlinearity, $\tilde{\alpha }=1$.

Minimizing Eq. 2 yields the phase diagram shown in Fig. 3B. The discontinuous shape transitions indicated by solid lines correspond to bistable configurations in the effective energy $\tilde{F}(\lambda )$ (Fig. 3C). Increasing active driving destabilizes spheres, but now, the elastic nonlinearity $\tilde{\alpha }$ controls whether the resulting shape is a worm or a pancake. In the neo-Hookean limit $\tilde{\alpha }\to 0$, the preferred shape is a compressed pancake. Intuitively, this corresponds to maximizing surface area $\tilde{A}$ without any elastic effects (Fig. 3C, left). For sufficiently large $\tilde{\alpha }$, the preferred shape is instead an elongated worm (Fig. 3C, middle), reflecting the bias toward uniaxial elongation over compression encoded in the Mooney-Rivlin theory (*64*). These worms can undergo a second snap-through transition (Fig. 3C, right), morphing to pancakes as active driving $|{\tilde{\gamma }}_{R}|$ is further increased or as the nonlinearity $\tilde{\alpha }$ is tuned.

In Fig. 3 (D and E), we demonstrate these continuum predictions in a microscopic model of active elastocapillarity, as shown in movie S1. We simulate the deformation of a spherical mesh of bulk nonlinear springs, coupled to surface springs exerting active stresses, as shown in Fig. 3D (see the “Numerics” section for details). At a critical active stress, the meshed solid destabilizes, with the resulting shape tunable via spring-level nonlinearity. By tuning the springs such that $\tilde{\alpha }$ crosses the critical value ${\tilde{\alpha }}^{*}$, we realize worms (Fig. 3D, top), pancakes, and snap-through transitions (Fig. 3D, bottom), in agreement with analytical predictions. In Fig. 3E, we compare the continuum theory Eq. 2 with numerical data for the stretch factor λ. Significantly, the quantitative theory-simulation agreement near the transition hints at universality.

The three transition lines in Fig. 3B meet at a critical point. This critical point is a direct consequence of the symmetry of the initial shape. In contrast to a sphere, an initial uniaxial anisotropy λ_0_ causes the critical point to split (Fig. 3F). For example, the phase diagram shown in Fig. 3F (top right) demonstrates how an initially elongated shape with λ_0_ > 1 can smoothly extend as active driving is increased. However, to compress into a pancake-like shape, the object must still cross a snap-through transition. The converse is true for an initially compressed shape (Fig. 3F, bottom right). In activity-anisotropy space, a cut through the critical point reveals an Ising-like transition (Fig. 3F, bottom left), with $-|{\tilde{\gamma }}_{R}|$ playing the role of temperature and λ_0_ playing the role of an external field. This observation motivates a universal characterization of shape transitions near the critical point using Landau theory.

In the above discussion, we introduced the Mooney-Rivlin model as a minimal choice. Other nonlinear elasticities will yield quantitatively different phase diagrams. However, near the critical point, the complete behavior of the active solid can be understood using symmetry-based arguments. For any initial shape, the Landau expansion guarantees that the effective free energy has the form

$$\tilde{F}(\epsilon )=r\epsilon^{2}-w\epsilon^{3}+u\epsilon^{4}-h\epsilon$$ (3)

where the linearized strain ϵ(=λ − 1) plays the role of an order parameter, the control parameter $r∼\Delta {\tilde{\gamma }}_{R}$ probes the distance to linear instability, $w(∼\Delta \tilde{\alpha }$ within the Mooney-Rivlin model) is the lowest-order nonlinear term, and *u* > 0 guarantees stability. The linear term *h* ∼ λ_0_ − 1 captures the effects of either shape anisotropy or external uniaxial stresses and is absent for cubes, spheres, and other spherical tops (i.e., shapes with an isotropic moment-of-inertia tensor). As a result, a critical point is generically expected for these symmetric shapes, with three weakly discontinuous transitions emanating from it, as in Fig. 3B. Although Landau theory breaks down at higher strains, this critical point controls the entire phase diagram shape.

We derive expressions for parameters *r*, *w*, *u*, and *h* within the Mooney-Rivlin model in the “Nonlinear theory” section. However, we emphasize that the form of Eq. 3 is fully constrained by symmetry. Hence, we expect the qualitative structure of our results to hold for a general nonlinear elasticity; the Landau theory presents a universal classification across all elastocapillary materials and shapes. The essential feature is simply that nonlinear effects appear in *w*, as is generically the case. In the Supplementary Materials, we explore the phase diagram of the Gent model of rubber elasticity (*66*, *67*), a singular example where nonlinearities only appear at quartic order.

## DISCUSSION

Active elastocapillarity couples field theories of different dimensionalities toward new materials design principles. Here, we have adopted a continuum approach applicable across systems, using linear elasticity to describe shape selection and wave propagation analytically. We have found quantitative agreement between continuum nonlinear elasticity and particle-based numerics in predicting both the final shape of active elastocapillary solids and snap-through transitions between them. Landau theory explains this agreement in terms of universal behavior about a critical point, allowing a classification based solely on the initial symmetry of the solid.

We have focused on the minimal case of a passive elastic solid, coupled to active stresses in the form of an effectively negative surface tension ϒ*_ij_* = −∣γ∣δ*_ij_*. The concept of active elastocapillarity may include a broader range of phenomena in which either bulk or surface stresses contain active components. While our attention has been on synthetic realizations, some of these ideas may also be relevant across living systems where surface stresses affect shape. Examples of biological phenomena with surface growth and (2D or 3D) elasticity include cellular symmetry breaking (*4*), buckling of thin actin shells (*37*), cellular layers (*36*), tissues (*42*, *44*, *45*), and modified wetting via differential growth (*68*). In contrast to the complexity of these systems, here, we have highlighted how minimal ingredients such as isotropic surface stresses and bulk elastic nonlinearity can already be used to design unexpected 3D metamaterial functionality.

The instabilities that we have uncovered, from snap-through to smooth deformations, suggest active elastocapillarity as a portable mechanism to achieve complex reconfigurable shapes. At the macroscale, we envision the design of soft robotic arms composed of anelastic backbone covered in simple actuators (Fig. 1C). Scaling active elastocapillarity down to soft nanoparticles (Fig. 1B), for which no reliable shape-control mechanism exists, may prove useful for applications ranging from drug delivery to self-assembly of photonic crystals.

## MATERIALS AND METHODS

### Linear instability and shape change in an active elastocapillary droplet

In this section, we derive the dispersion relation and linear instabilities of an active elastocapillary sphere. Recently, Tamim and Bostwick (*57*) have given an analysis of the vibrations of the passive case, extending classical results for the purely elastic (*69*) and capillary limits (*70*). Here, we instead consider the active case in which the surface terms consist of both a negative surface tension γ and bending modulus κ. The approach is to take a bulk ansatz satisfying the equations of linear elastodynamics (*34*) and impose a matching boundary condition between bulk and surface stresses. This boundary condition leads to a solvability criterion, whose solution gives the dispersion and regimes of linear instability.

We first derive the boundary condition, balancing active stresses with restoring elasticity. Given a (2D) surface with surface tension γ and bending rigidity κ, a variation of the Helfrich surface free energy

$$F=\int \mathrm{dA}[2\kappa {\left(H-c_{0}\right)}^{2}+\gamma ]$$ (4)

gives the shape equation for vesicles (*65*)

$$P=2\kappa [\nabla^{2}H+2(H-c_{0})(H^{2}-K+c_{0}H)]-2\gamma H$$ (5)

where *P* is (minus) the normal component of the bulk stress tensor, *H* is the mean curvature of the solid’s boundary, *K* is the Gaussian curvature, and *c*_0_ allows for a preferred nonzero mean curvature. We now expand *H*, *K*, and *P* to first order about a spherical shape, considering a normal perturbation ψ***n***, where ***n*** is the outward unit normal

$$\begin{array}{c} H=H_{0}+\delta H+O(\psi^{2})=-\frac{1}{R}+\delta H+O(\psi^{2})\\ K=K_{0}+\delta K+O(\psi^{2})=\frac{1}{R^{2}}+\delta K+O(\psi^{2})\\ P=P_{0}+\delta P+O(\psi^{2}) \end{array}$$ (6)

Here, δ*H* and δ*K* are given by (*71*, *72*)

$$\begin{array}{c} \delta H=\frac{1}{R^{2}}\psi +\frac{1}{2}\nabla^{2}\psi \\ \delta K=-\frac{2}{R^{3}}\psi -\frac{1}{R}\nabla^{2}\psi \end{array}$$ (7)

Substituting Eqs. 6 and 7 into the shape equation Eq. 5 yields the normal component of the stress matching condition

$$-\sigma_{\mathrm{nn}}=-2[(\gamma +2\kappa \;c_{0}(\frac{2}{R}+c_{0}))-\kappa \nabla^{2}]\delta H$$ (8)

with the tangential component σ_τ*n*_ = 0, where τ denotes the surface tangent. For an expansion in terms of spherical harmonics, we take $\psi =NY_{l}^{m}=NP_{l}^{m}(\text{cos}\;\theta )e^{\mathrm{im}\phi }$, with $P_{l}^{m}$ as the associated Legendre polynomial and *N* as a normalization factor (*73*). Then, ∇^2^ψ = − *l*(*l* + 1)ψ and Eq. 8 simplifies to

$$-\sigma_{\mathrm{nn}}=[\gamma +2\kappa \;c_{0}(\frac{2}{R}+c_{0})+\frac{\kappa }{R^{2}}l(l+1)]\frac{\left(2-l(l+1)\right)}{R^{2}}\psi$$ (9)

From Eqs. 8 and 9, we see the effect of the bending modulus and spontaneous curvature is to shift γ as

$$\gamma \to \gamma +2\kappa \;c_{0}(\frac{2}{R}+c_{0})+\frac{\kappa }{R^{2}}l(l+1)$$ (10)

Two natural special cases of this result are *c*_0_ = 0 (no spontaneous curvature) and *c*_0_ = − 1/*R* (spontaneous curvature matching the initial mean curvature *H*_0_). Here, we focus on the case *c*_0_ = 0, for which Eq. 8 simplifies to Eq. 1. However, note that only the last term in Eq. 10 depends on *l* and the effect of *c*_0_ can be completely reabsorbed into the effective surface tension γ.

With the mapping of Eq. 10, the problem reduces to that considered in (*57*). The bulk stress tensor σ is given by the general solution to the linear elastodynamic equations in spherical coordinates (*69*). Substituting this solution into Eq. 9 gives the solvability condition, which we invert numerically to obtain the dispersion. Below, we state this solvability condition for the limit of an incompressible material, in terms of dimensionless variables coming from the sphere radius *R* and associated timescale $\tau_{R}=R/\sqrt{\mu /\rho }$

$$\begin{array}{c} l=\mathrm{qR}\\ {\tilde{\omega }}_{R}=\tau_{R}\omega \\ {\tilde{\gamma }}_{R}=\frac{l_{\gamma }}{R}\\ {\tilde{\kappa }}_{R}=\frac{\kappa }{\mu R^{3}}={\left(\frac{l_{\kappa }}{R}\right)}^{3} \end{array}$$ (11)

In terms of these dimensionless quantities, the dispersion is given by the solution of

$$\begin{array}{c} {\tilde{\omega }}_{R}[2+{\tilde{\omega }}_{R}^{2}-l^{3}({\tilde{\gamma }}_{R}+{\tilde{\kappa }}_{R}l(l+1))+2l(1+{\tilde{\gamma }}_{R}+{\tilde{\kappa }}_{R}l(l+1))-l^{2}\\ \left(4+{\tilde{\gamma }}_{R}+{\tilde{\kappa }}_{R}l(l+1))]j_{l}({\tilde{\omega }}_{R})-2[{\tilde{\omega }}_{R}^{2}+l(2+{\tilde{\gamma }}_{R}+{\tilde{\kappa }}_{R}l(l+1)\right)\\ (2-l-l^{2})]j_{l+1}({\tilde{\omega }}_{R})=0 \end{array}$$ (12)

where *j_l_* is a spherical Bessel function of the *l*th kind.

Figure 2 (B and C) was found by solving Eq. 12 numerically. Note that the dispersion Eq. 12 has an infinite number of branches, corresponding to the roots of *j_l_*, spheroidal modes ω*_R_*(*s*, *l*) are indexed by a radial “quantum number” *s* and polar wave number *l*, being degenerate with respect to the azimuthal wave number *m*. Only the *s* = 1 branch couples to the instability described here, and it is this branch that is shown in Fig. 2 (B and C).

### Waves and instabilities in an active elastocapillary half-space

In this section, we derive the spectrum of the linearized equations of motion for an active elastocapillary half-space. These results follow from the *l* → ∞ limit of the “Linear instability and shape change in an active elastocapillary droplet” section, in particular Eq. 12. However, an independent derivation in the planar case has the virtue of being much simpler than the spherical case, and we extend it to study the effects of viscosity and bulk dispersion in the Supplementary Materials. Passive elastocapillary waves have been studied from the perspective of a viscous fluid (*59*) or an elastic solid. Here, we take the elastic solids perspective (*58*) and consider the active case in which a negative surface tension γ is regularized in the high wave number limit by a bending modulus κ. Our approach applies equally to 2D or 3D materials.

Before giving a detailed argument, basic scaling considerations capture the main phenomenology. Consider a slab of material of undeformed surface area *A* with a deformed surface height *h*(*x*). The energy stored in surface deformations is $E_{s}∼(\frac{\gamma }{2}h^{\prime }{\left(x\right)}^{2}+\frac{\kappa }{2}h\prime \prime {\left(x\right)}^{2})A$, and the bulk energy *E_b_* ∼ μ*h*′(*x*)^2^*Al*, where *l* is the depth that surface deformations penetrate into the bulk. Assuming *l* ∼ 1/*q*, the total energy per unit volume is then $E/(\mathrm{lA})∼(\mu +\frac{\gamma }{2}q+\frac{\kappa }{2}q^{4})h^{2}$, and we see that γ acts as a *q*-dependent shift to the shear modulus, $\mu (q)=\mu +\frac{\gamma }{2}q$. As *q* increases, for γ < 0, μ(*q*) softens, with higher wave numbers feeling progressively weaker elastic restoring forces. At *q* ∼ 1/*l*_γ_, restoring elasticity vanishes entirely, with κ regularizing high wave numbers. We thus expect the threshold wave number for instability to scale as 1/*l*_γ_.

We now give a detailed derivation of the spectrum. We consider a half-space *z* ≤ 0. The equations of linear elastodynamics in the bulk material are (*34*)

$$\rho \ddot{u_{i}}=\partial_{j}\sigma_{\mathrm{ij}}$$ (13)

where ρ is the density and *u_i_* is the displacement. For an isotropic material, the stress tensor $\sigma_{\mathrm{ij}}=B\delta_{\mathrm{ij}}u_{\mathrm{kk}}+2\mu (u_{\mathrm{ij}}-\frac{1}{d}u_{\mathrm{kk}}\delta_{\mathrm{ij}})$, where $u_{\mathrm{ij}}=\frac{1}{2}(\partial_{i}u_{j}+\partial_{j}u_{i})$ is the linearized strain and *d* is the spatial dimension. Equation 13 supports longitudinal and transverse waves, of wave vector *q* and frequency ω, propagating along *x* and decaying as *z* → − ∞ (*34*, *58*)

$$\begin{array}{c} u_{L}=(qe_{x}-i\alpha_{L}e_{z})\text{exp}\;[i(\mathrm{qx}-\omega t)+\alpha_{L}z]\\ u_{T}=(i\alpha_{T}e_{x}+qe_{z})\text{exp}\;[i(\mathrm{qx}-\omega t)+\alpha_{T}z] \end{array}$$ (14)

Here, $\alpha_{T}=\sqrt{q^{2}-\rho \omega^{2}/\mu }$ and $\alpha_{L}=\sqrt{q^{2}-\rho \omega^{2}/M}$ are the inverse penetration depths along *z*, with the longitudinal modulus $M=B+2\frac{\mu (d-1)}{d}$. A general displacement **u** = *u_x_***e***_x_* + *u_z_***e***_z_* is written as

$$\begin{array}{c} u=[A_{L}(qe_{x}-i\alpha_{L}e_{z})\text{exp}\;\alpha_{L}z+A_{T}(i\alpha_{T}e_{x}+qe_{z})\text{exp}\\ \alpha_{T}z]\text{exp}\;i(\mathrm{qx}-\omega t) \end{array}$$ (15)

We now take the bulk ansatz Eq. 21 and substitute it into a stress matching boundary condition at *z* = 0. The boundary has surface tension γ, bending modulus κ, and effective free energy

$$F=\int \mathrm{dx}[\frac{\gamma }{2}{\left(\frac{\mathrm{dh}}{\mathrm{dx}}\right)}^{2}+\frac{\kappa }{2}{\left(\frac{d^{2}h}{dx^{2}}\right)}^{2}]$$ (16)

where *h*(*x*) is the height of the free surface above *z* = 0. Matching the *z* component of bulk displacement *u_z_* to height *h* yields the stress matching condition

$$\begin{array}{cc} {\sigma_{\mathrm{zz}}|}_{z=0} & =\gamma \frac{d^{2}u_{z}}{{\mathrm{dz}}^{2}}-\kappa \frac{d^{4}u_{z}}{{\mathrm{dz}}^{4}}\\ {\sigma_{\mathrm{xx}}|}_{z=0} & =0 \end{array}$$ (17)

Substituting Eq. 15 into Eq. 17 gives

$$[\begin{array}{cc} {}[-i\alpha_{L}^{2}M+iq^{2}(M-2\mu )]-i\alpha_{L}(\gamma +\kappa q^{2})q^{2} & 2\mu q\alpha_{T}+(\gamma +\kappa q^{2})q^{3}\\ 2\alpha_{L}q & i(\alpha_{T}^{2}+q^{2}) \end{array}][\begin{array}{c} A_{L}\\ A_{T} \end{array}]=0$$ (18)

At this point, we take the incompressible limit: as *B*, *M* → ∞, α*_L_* → *q*, and Eq. 18 simplifies to

$$[\begin{array}{cc} -i(2\mu q^{2}+(\gamma +\kappa q^{2})q^{3}) & 2\mu q\alpha_{T}+(\gamma +\kappa q^{2})q^{3}\\ 2q^{2} & i(\alpha_{T}^{2}+q^{2}) \end{array}[\begin{array}{c} A_{L}\\ A_{T} \end{array}]=0$$ (19)

The dispersion is obtained from the solvability condition that the determinant of Eq. 19 must vanish

$$\rho \omega^{2}-\frac{4\mu q^{2}\alpha_{T}}{q+\alpha_{T}}-\gamma q^{3}-\kappa q^{5}=0$$ (20)

Equation 20 reduces to the passive elastocapillary dispersion (in the incompressible limit) for γ > 0 and κ → 0 (*58*), and the standard Rayleigh wave dispersion for κ, γ → 0 (*34*). Intrinsic length and time scales are given by

$$\begin{array}{c} l_{\gamma }=\frac{\left|\gamma \right|}{\mu }\;(\text{elastocapillary length})\\ l_{\kappa }={\left(\frac{\kappa }{\mu }\right)}^{\frac{1}{3}}\;(\text{bendoelastic length})\\ \tau_{\gamma }=\frac{l_{\gamma }}{\sqrt{\mu /\rho }}=\sqrt{\frac{{\rho \gamma }^{2}}{\mu^{3}}}(\text{elastocapillary time}) \end{array}$$ (21)

which we use to define nondimensionalized variables

$$\begin{array}{c} {\tilde{q}}_{\gamma }=l_{\gamma }q\\ {\tilde{\omega }}_{\gamma }=\tau_{\gamma }\omega \\ {\tilde{\alpha }}_{\gamma }=\sqrt{{\tilde{q}}_{\gamma }^{2}-{\tilde{\omega }}_{\gamma }^{2}}\\ {\tilde{\kappa }}_{\gamma }={\left(\frac{l_{\kappa }}{l_{\gamma }}\right)}^{3}=\frac{{\kappa \mu }^{2}}{{\left|\gamma \right|}^{3}} \end{array}$$ (22)

In dimensionless form, Eq. 20 is then

$${\tilde{\omega }}_{\gamma }^{2}-\frac{4{\tilde{q}}_{\gamma }^{2}{\tilde{\alpha }}_{\gamma }}{{\tilde{q}}_{\gamma }+{\tilde{\alpha }}_{\gamma }}-\text{sgn}(\gamma ){\tilde{q}}_{\gamma }^{3}-{\tilde{\kappa }}_{\gamma }{\tilde{q}}_{\gamma }^{5}=0$$ (23)

Here, we focus on the case of negative surface tension, sgn(γ) = −1. The phase diagrams shown in Fig. 2 (D and E) and the dispersions shown in Fig. 2F are found by solving Eq. 23 numerically. However, the threshold at which instability occurs can be found analytically: letting ${\tilde{\omega }}_{\gamma }=0$ in Eq. 23 gives the condition

$${\tilde{q}}_{\gamma }^{2}(-\kappa {\tilde{q}}_{\gamma }^{3}+{\tilde{q}}_{\gamma }-2)=0$$ (24)

We require the cubic in Eq. 24 to have degenerate roots, as must be the case at instability. This gives values for the wave number ${\tilde{q}}_{\gamma }^{*}$ and dimensionless bending modulus ${\tilde{\kappa }}_{\gamma }^{*}$ at which instability sets in

$$\begin{array}{c} {\tilde{q}}_{\gamma }^{*}=3\\ {\tilde{\kappa }}_{\gamma }^{*}={\left(\frac{1}{3}\right)}^{3} \end{array}$$ (25)

We may compare the structure of the above derivation to the intuitive argument presented at the beginning of this section. To the extent that α*_T_* ≈ *q* (strictly true in the Rayleigh wave limit *q* → 0), Eq. 19 shows that we can indeed formally absorb the effects of surface tension into a *q*-dependent shear modulus, $\mu (q)=\mu +\frac{\gamma }{2}q$. Furthermore, referring to Eq. 25, we find that the threshold wave number for instability occurs at *q* ∼ 1/*l*_γ_, as expected.

### Nonlinear theory

#### Derivation of the Mooney-Rivlin energy

In this section, we detail the derivation of Eq. 2, giving expressions for ${\tilde{F}}_{\text{NH}}$, ${\tilde{F}}_{\text{MR}}$, ${\tilde{\gamma }}_{R}\tilde{A}$, and ${\tilde{F}}_{\text{bend}}$ for the case of a uniaxial ellipsoid. We emphasize at the outset that the details of the results depend on the ellipsoidal geometry that we have chosen, but their structure does not. One may repeat these calculations for other starting geometries, for example, a cube, and obtain similar results.

Elastic deformations are described by the deformation gradient tensor Λ (*74*). For a 3D material, $\Lambda \equiv \frac{\partial X}{\partial x}$ is a 3D tensor that gives the local mapping of material points from the undeformed state **x** to the deformed state **X**. In general, Λ depends on **x**, the position within the undeformed state. Here, we assume a homogeneous deformation, for which Λ is constant. Given Λ, the three lowest-order rotational invariants that can appear in the elastic free energy density *f*_elastic_ are *I*_1_ = Tr*C*, $I_{2}=\frac{1}{2}{\left(\text{Tr}C\right)}^{2}-\text{Tr}(C^{T}C)$, *I*_3_ = Det*C*, where *C* = Λ*^T^*Λ is the right Cauchy-Green deformation tensor (*74*). *I*_1_ is the neo-Hookean term, and *I*_3_ describes volumetric deformations, i.e., *I*_3_ = 1 for incompressible solids, as we consider here. *I*_2_ is the Mooney-Rivlin term, often used to phenomenologically account for material nonlinearity in rubbers (*64*). The elastic part of the free energy density can be written as

$$f_{\text{elastic}}=c_{1}I_{1}+c_{2}I_{2}+\cdots$$ (26)

We consider a uniaxial deformation, $\Lambda =\text{diag}(1/\sqrt{\lambda },1/\sqrt{\lambda },\lambda )$, and let $\mu =\frac{1}{2}(c_{1}+c_{2})$ [μ is indeed the linear elastic shear modulus (*74*)], $\alpha =\frac{1}{2}(c_{1}-c_{2})$. Equation 26 is then

$$f_{\text{elastic}}=\frac{\mu -\alpha }{2}(\frac{2}{\lambda }+\lambda^{2})+\frac{\alpha }{2}(\frac{1}{\lambda^{2}}+2\lambda )$$ (27)

For a uniaxial ellipsoid of radii $\left(R/\sqrt{\lambda },R/\sqrt{\lambda },R\lambda \right)$ the total elastic free energy is

$$F_{\text{elastic}}=\frac{4\pi }{3}\mu [\frac{1-\tilde{\alpha }}{2}(\frac{2}{\lambda }+\lambda^{2})+\frac{\tilde{\alpha }}{2}(\frac{1}{\lambda^{2}}+2\lambda )]R^{3}$$ (28)

where we identify the first term in Eq. 28 as *F*_NH_ and the second as *F*_MR_.

The surface energy is

$$\gamma A_{\text{ellipsoid}}=\frac{2\pi \gamma }{\lambda }[1+\frac{\lambda^{\frac{3}{2}}}{e(\lambda )}\text{arcsin}\;e(\lambda )]R^{2}$$ (29)

where $e(\lambda )=\sqrt{1-\lambda^{-3}}$ is the eccentricity. For the bending energy *F*_bend_, we use the Helfrich form discussed in the “Linear instability and shape change in an activeelastocapillary droplet” section

$$F_{\text{bend}}=2\kappa \int \mathrm{dA}{\left(H-c_{0}\right)}^{2}$$ (30)

We now evaluate Eq. 30 for the case of a uniaxial ellipsoid. Finite *c*_0_ does not qualitatively change the structure of our results, and we consider *c*_0_ = 0 for simplicity. The area element is

$$\mathrm{dA}=\frac{R^{2}}{\sqrt{2}}{\left(\frac{1}{\lambda^{2}}+\lambda +(\frac{1}{\lambda^{2}}-\lambda )\text{cos}\;2v\right)}^{\frac{1}{2}}\text{sin}\;v\;\text{dudv}$$ (31)

where *u* and *v* are the azimuthal and polar angles on the ellipsoid. The mean curvature *H* is

$$H=\frac{3+\lambda^{3}-(\lambda^{3}-1)\text{cos}\;2v}{\sqrt{2}R\lambda {\left(\frac{1}{\lambda^{2}}+\lambda +(\frac{1}{\lambda^{2}}-\lambda )\text{cos}\;2v\right)}^{\frac{3}{2}}}$$ (32)

Equation 30 then simplifies to

$$F_{\text{bend}}=4\pi \kappa \int_{0}^{\pi }\mathrm{dv}\frac{{\left(3+\lambda^{3}-(\lambda^{3}-1)\text{cos}\;2v\right)}^{2}\text{sin}\;v}{2\sqrt{2}\lambda^{2}{\left(\frac{1}{\lambda^{2}}+\lambda +(\frac{1}{\lambda^{2}}-\lambda )\text{cos}\;2v\right)}^{\frac{5}{2}}}$$ (33)

which may be evaluated exactly; the result is

$$F_{\text{bend}}=\frac{2\pi \kappa }{3}(\frac{2}{\lambda^{3}}+\frac{3\lambda^{3}{\text{tanh}}^{-1}(\sqrt{1-\lambda^{3}})}{\sqrt{1-\lambda^{3}}}+7)$$ (34)

Combining Eqs. 28, 29, and 34, all divided by μ*R*^3^, gives the total free energy Eq. 2.

#### Landau theory coefficients for the Mooney-Rivlin model

Here, we argued that the behavior of an active elastocapillary sphere near the critical point λ = 1 can be understood only on the basis of symmetries using the Landau expansion Eq. 3. We now derive the coefficients *r*, *w*, and *u* of Eq. 3 for the case of the Mooney-Rivlin free energy Eq. 2. Expanding Eqs. 28, 29, and 34 in the strain ϵ (=λ − 1), we obtain

$$\begin{array}{c} \frac{F}{\mu R^{3}}=A_{2}\epsilon^{2}+A_{3}\epsilon^{3}+A_{4}\epsilon^{4}+\ldots \\ A_{2}=\frac{2}{5}\pi (4{\tilde{\gamma }}_{R}+24{\tilde{\kappa }}_{R}+5)\\ A_{3}=-\frac{4\pi }{105}(35\tilde{\alpha }+52{\tilde{\gamma }}_{R}+360{\tilde{\kappa }}_{R}+35)\\ A_{4}=\frac{2}{105}\pi (105\tilde{\alpha }+110{\tilde{\gamma }}_{R}+1056{\tilde{\kappa }}_{R}+70) \end{array}$$ (35)

Equation 35 has a critical point at ${\tilde{\gamma }}_{R}^{*}$, ${\tilde{\alpha }}^{*}$, ${\tilde{\kappa }}_{R}^{*}$, where

$$\begin{array}{c} {\tilde{\gamma }}_{R}^{*}=-\frac{1}{4}(5+24{\tilde{\kappa }}_{R}^{*})\\ {\tilde{\alpha }}^{*}=\frac{6}{35}(5-8{\tilde{\kappa }}_{R}^{*}) \end{array}$$ (36)

Expanding as $\Delta \gamma ={\tilde{\gamma }}_{R}-{\tilde{\gamma }}_{R}^{*}$, $\Delta \alpha =\tilde{\alpha }-{\tilde{\alpha }}^{*}$, and $\Delta \kappa ={\tilde{\kappa }}_{R}-{\tilde{\kappa }}_{R}^{*}$, we obtain the structure of the free energy about this critical point

$$\begin{array}{c} \frac{F}{\mu R^{3}}=r\epsilon^{2}+w\epsilon^{3}+u\epsilon^{4}+\ldots \\ r=\frac{8}{5}\pi (\Delta \gamma +6\Delta \kappa )\\ u=\frac{1}{105}\pi (-140\Delta \alpha -208\Delta \gamma -1440\Delta \kappa )\\ w=\frac{1}{105}\pi (504{\tilde{\kappa }}_{R}^{*}+45) \end{array}$$ (37)

where we omit terms such as Δγϵ^4^.

**Fig. 4.**
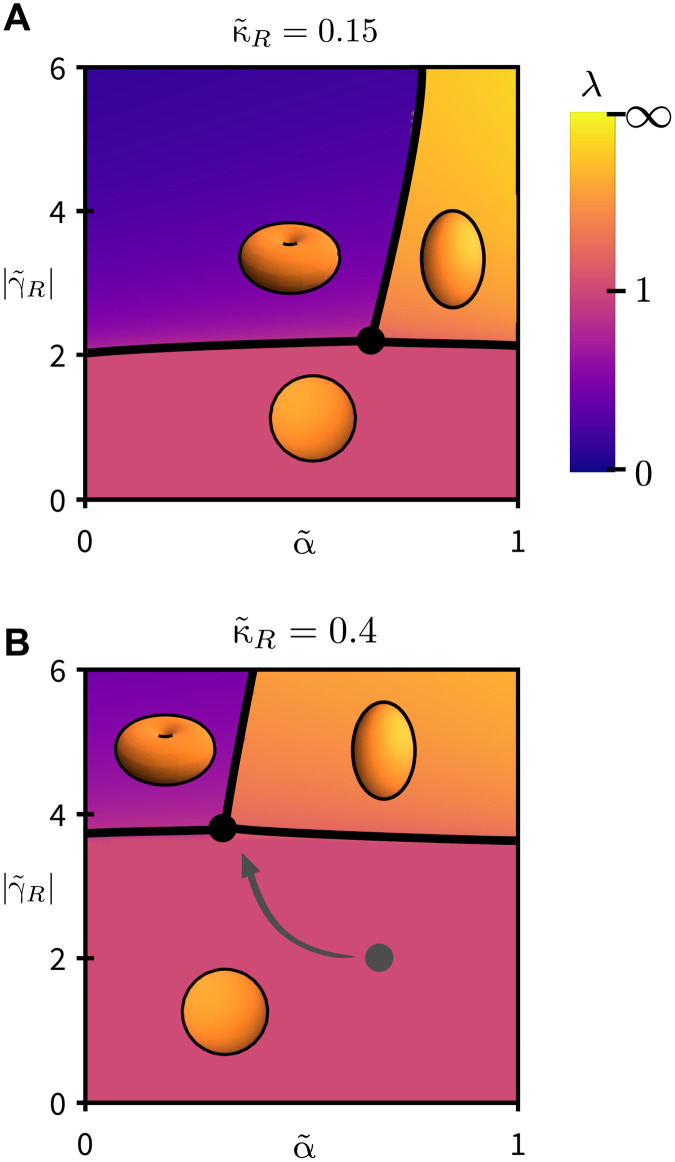
Structure of the worm/pancake phase diagram. (**A**) Three weakly discontinuous transitions, described by the Landau theory Eq. 37, meet at a critical point given by Eq. 36. (**B**) As the bending modulus ${\tilde{\kappa }}_{R}$ increases, the critical point is driven to larger active driving $\left|{\tilde{\gamma }}_{R}\right|$ and lower material nonlinearity $\tilde{\alpha }$, enlarging the worm-like region of parameter space.

In Fig. 4, we show phase diagrams obtained from minimizing the exact free energy Eq. 2. We can interpret their structure in light of Eqs. 36 and 37, with a focus on the interplay between bulk elasticity and surface effects. The Landau theory of Eq. 37 corresponds to three weakly discontinuous transitions in the $\tilde{\alpha },|{\tilde{\gamma }}_{R}|$ plane, meeting at a critical point (Fig. 4A). This structure is unchanged by varying the bending modulus ${\tilde{\kappa }}_{R}$. However, increasing the bending modulus drives the critical point to higher values of active driving $\left|{\tilde{\gamma }}_{R}\right|$ and lower material nonlinearity $\tilde{\alpha }$ (Fig 4B), enlarging the region of phase space in which worms are favored over pancakes. In this sense, both material nonlinearity $\tilde{\alpha }$ and bending rigidity ${\tilde{\kappa }}_{R}$ conspire to produce worm-like structures, as opposed to the more intuitively obvious pancake.

### Numerics

In this section, we describe the microscopic ball-spring model and numerical methods used to realize the predictions of the continuum theory shown in Fig. 3.

#### Microscopic model

**Fig. 5.**
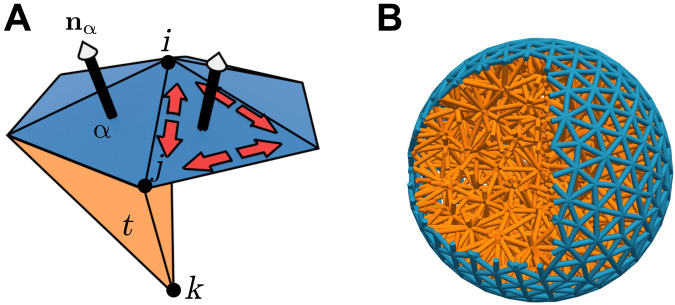
Microscopic ball-spring model. (**A**) Schematic of the microscopic model at the surface of the meshed ball, showing vertices *i*, *j*, *k*…, edges *ij*, *jk*…, surface triangular plaquettes α, and volume tetrahedra *t*. (**B**) Cut-through of the meshed ball used in simulation, showing prestressed surface springs (blue) and unstressed bulk springs (orange).

We first construct a disordered tetrahedral meshing of the ball, as shown in Fig. 5. We label the vertices *i*, edges *ij*, triangles α, and tetrahedra *t*. A microscopic energy for deformations of this mesh is given by a spring energy *F*_spring_, a surface bending energy *F*_bend_, and an approximate volume constraint *F*_vol_

$$F=F_{\text{spring}}+F_{\text{bend}}+F_{\text{bulk}}$$ (38)

For *F*_spring_, we place a spring along every edge *ij* of the mesh. These springs have length *r_ij_*, and rest length $r_{\mathrm{ij}}^{0}$, from which we define the extension $\lambda_{\mathrm{ij}}=r_{\mathrm{ij}}/r_{\mathrm{ij}}^{0}$. The spring energy is then

$$F_{\text{spring}}=\frac{k_{m}}{2}\sum_{i>j}(1-\alpha_{m})(2\lambda_{\mathrm{ij}}^{-1}+\lambda_{\mathrm{ij}}^{2})+\alpha_{m}(\lambda_{\mathrm{ij}}^{-2}+2\lambda_{\mathrm{ij}})$$ (39)

i.e., each spring acts as an incompressible Mooney-Rivlin solid with microscopic neo-Hookean constant *k_m_* and material nonlinearity α*_m_*. To implement dilational surface stresses, we prestress the springs on the surface of the ball, initializing them at an extension λ*_m_* < 1. The bulk springs are initialized at their rest length, λ = 1. We vary the macroscopic nonlinearity in material response $\tilde{\alpha }$ by varying α*_m_* for the bulk springs, keeping the surface springs at α*_m_* = 0. A bending energy *F*_bend_ is given by (*75*)

$$F_{\text{bend}}=\kappa_{m}\sum_{\alpha ,\beta }(1-n_{\alpha }\cdot n_{\beta })$$ (40)

where the sum is over neighboring triangular plaquettes α and β on the surface of the ball, with **n**_α_ as the normal to plaquette α. Last, we approximately enforce incompressibility with an additional energetic penalty on volume changes of the tetrahedra *t* of the mesh (*74*)

$$F_{\text{vol}}=B_{m}\sum_{t}{\left(V_{t}-V_{t}^{0}\right)}^{2}$$ (41)

where *V_t_* is the current volume of a tetrahedron and $V_{t}^{0}$ is its rest volume.

The microscopic energy Eq. 38 contains *k_m_*, α*_m_* κ*_m_*, λ*_m_*, and *B_m_* as microscopic parameters. We now describe a mapping to the continuum shear modulus μ, bulk modulus *B*, material nonlinearity $\tilde{\alpha }$, bending rigidity κ, and surface tension γ. Given a typical mesh length scale *a*, dimensional analysis gives

$$\mu ∼\frac{k_{m}}{a^{3}}$$ (42)

$$\tilde{\alpha }∼\alpha_{m}$$ (43)

$$B∼B_{m}a^{3}$$ (44)

For an analytical estimate of the relation between κ*_m_* and κ, we may calibrate using the continuum limit of the discrete bending energy Eq. 40 for a sphere, $4\pi \kappa_{m}/\sqrt{3}$ (*75*). Comparing this to the continuum energy 8πκ gives the relation

$$\kappa =\frac{1}{2\sqrt{3}}\kappa_{m}$$ (45)

For an estimate of the mapping from λ*_m_* to |γ|, one can show that the energy per unit area of a triangular spring mesh of side length λ*_m_a*, composed of neo-Hookean springs, is given by

$$\left|\gamma |=\frac{2\sqrt{3}}{a^{2}}k_{m}(1+\frac{2}{\lambda_{m}^{3}}\right)$$ (46)

#### Numerical methods

The data in Fig. 3 are generated by numerically minimizing Eq. 38 at fixed *k_m_*, α*_m_*, κ*_m_*, and *B_m_*, with λ*_m_* progressively decreasing from λ*_m_* = 1 (giving progressively stronger dilational surface stresses). The final state of each minimization is then used as an initialization condition for the next. The minimizer used is the SciPy implementation of Broyden–Fletcher–Goldfarb–Shanno (BFGS) algorithm, with mesh vertex coordinates as input and gradient norm stopping threshold of 10^−2^. For the data shown in Fig. 3, a ball of radius *R* = 1 (which defines the arbitrary spatial unit) is meshed with typical edge spacing *a* = 0.2. Microscopic parameters *k_m_* = 0.013, *B_m_* = 50,000, and κ*_m_* = 2.5 are fixed for all runs. The three curves in Fig. 3E correspond to microscopic nonlinearities α*_m_* = 0 (green triangles), 0.3 (blue circles), and 0.4 (orange squares). From the numerical data, we first map λ*_m_* to γ using Eq. 46. To obtain the values of λ shown in Fig. 3E, an ellipsoid is then least-squares fit to the boundary vertices of the numerically relaxed mesh. The fit returns three ellipsoid axes, two of which are of similar magnitude (δλ/λ < 0.1), the third of which defines λ.

Last, we use the location of the critical point to fit the continuum theory Eq. 2 to this data, with ${\tilde{\kappa }}_{R}$, $\tilde{\alpha }$ as fitting parameters. The theoretical fit shown in Fig. 3 corresponds to ${\tilde{\kappa }}_{R}=0.3$, $\tilde{\alpha }={\tilde{\alpha }}^{*}$ via Eq. 36. An independent assessment of ${\tilde{\kappa }}_{R}$ may be made using Eqs. 45 and 44, from which we estimate κ ≈ 0.72, μ ≈ 5 (independent measurement of μ for the meshed sphere used in simulation places μ ≈ 4). These estimates give ${\tilde{\kappa }}_{R}\approx 0.2$, consistent with the value that we obtain from our fit.

## Supplementary Materials

### This PDF file includes:

Feature sharpening Modifications to active elastocapillarity: The effect of viscosity and bulk dispersion Active elastocapillarity and surface wrinkling Degenerate material nonlinearity: The Gent model Figs. S1 and S2
