# Supplementary material for: Active elastocapillarity in soft solids with negative surface tension
Source: Sci Adv. 2022 Mar 11;8(10):eabk3079. doi: 10.1126/sciadv.abk3079 (PMC8916726; doi:10.1126/sciadv.abk3079)
Supplement: Supplementary file 1 — Feature sharpening Modifications to active elastocapillarity: The effect of viscosity and bulk dispersion Active elastocapillarity and surface wrinkling Degenerate material nonlinearity: The Gent model Figs. S1 and S2 [file sciadv.abk3079_sm.pdf]

Science Advances
AAAS

Supplementary Materials for

Active elastocapillarity in soft solids with negative surface tension

Jack Binysh, Thomas R. Wilks, Anton Souslov*

*Corresponding author. Email: a.souslov@bath.ac.uk

Published 11 March 2022, *Sci. Adv.* **8**, eabk3079 (2022)
DOI: 10.1126/sciadv.abk3079

The PDF file includes:

Feature sharpening
Modifications to active elastocapillarity: The effect of viscosity and bulk dispersion
Active elastocapillarity and surface wrinkling
Degenerate material nonlinearity: The Gent model
Figs. S1 and S2
Legend for movie S1

Other Supplementary Material for this manuscript includes the following:

Movie S1

A. Feature sharpening

A positive surface tension minimises area, causing sharp edges and features to be rounded. Given a ridge [51, 53] or solid cone [55] of material, positive surface tension blunts the tip to a smoothed cap. In this section, we consider instead the effect of negative surface tension on a 3D cone. We find that, by contrast, surface area maximisation sharpens the cone to a cusped structure. Below, we first derive an analytic prediction for the profile of this cusp under the assumption of homogeneous deformation. We then demonstrate this sharpening effect in finite element elasticity simulations.

1. *Theoretical prediction for cusp profile*

Let us first consider the elongation of each cylindrical slice of the cone, and then integrate along the conical axis to find the resulting shape. Refs. [54, 55] consider the effect of surface tension γ on a cylinder made of neo-Hookean material, of radius ρ and shear modulus μ. The total energy of the cylinder is

$$E = E_{\text{elastic}} + \gamma A, \tag{S1}$$

where $E_{\text{elastic}} = 2\pi\mu\rho^2 \left[\left(\frac{1}{\lambda} - 1\right) + \frac{1}{2}(\lambda^2 - 1)\right]$ is the neo-Hookean elastic energy and $A = 2\pi\rho\sqrt{\lambda}$ is the curved surface area of the cylinder (excluding the caps). Minimizing with respect to λ, we find that surface tension distorts the cylinder along its axis by a factor λ, where

$$\lambda = \left[-\frac{\gamma}{4\mu\rho} + \sqrt{1 + \left(\frac{\gamma}{4\mu\rho}\right)^2}\,\right]^{\frac{2}{3}}. \tag{S2}$$

This result applies regardless of the sign of γ. For a negative surface tension ($\gamma < 0$), the stretch factor λ is greater than one ($\lambda > 1$), and the cylinder elongates. The volume is given by $\rho^2\lambda$, and so for the incompressible neo-Hookean material there is a corresponding radial contraction to a deformed radius $R \sim \rho/\sqrt{\lambda}$. Taking the thin cylinder limit $|\gamma|/\mu \gg \rho$ of Eq. (S2), for a negative surface tension we have a stretch factor $\lambda \sim (l_\gamma/\rho)^{\frac{2}{3}}$, where $l_\gamma = |\gamma|/\mu$ is the elastocapillary length.

Now, consider a three-dimensional cone of angle θ aligned along z (Fig. **1f**). Each cylindrical slice $z \to z + dz$ then experiences an elongation $\lambda(z) \sim (l_\gamma/\theta z)^{\frac{2}{3}}$. To find the total elongation, we integrate along the undeformed coordinate z to obtain the deformed height Z:

$$Z \sim \int_0^z \lambda(z)dz \sim \left(\frac{l_\gamma}{\theta}\right)^{\frac{2}{3}} z^{\frac{1}{3}}. \tag{S3}$$

We can express this result in terms of the deformed radius R:

$$R(z) \sim \frac{\theta z}{\sqrt{\lambda(z)}} \sim l_\gamma \left(\frac{\theta z}{l_\gamma}\right)^{\frac{4}{3}}. \tag{S4}$$

Substituting Eq. (S4) into Eq. (S3) gives

$$Z \sim \frac{l_\gamma}{\theta}\left(\frac{R}{l_\gamma}\right)^{\frac{1}{4}}, \tag{S5}$$

where now $Z(R)$ is the height of the deformed cone as a function of its deformed radius. The exponent in Eq. (S5) is less than 1, indicating a cusped structure, which becomes more pronounced with decreasing conical angle θ.

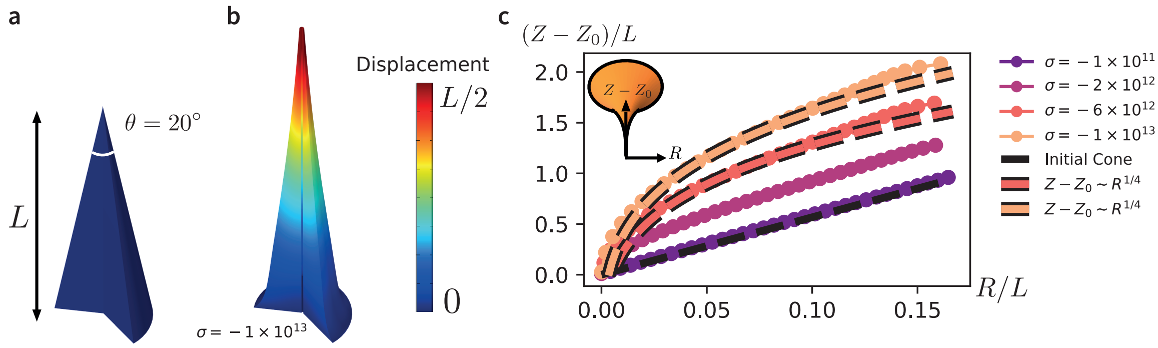

FIG. S1: **Sharpening of an active elastocapillary cone to a cusp**. **a–b.** A 3D elastic cone sharpens and elongates to a cusped structure under the action of dilational surface stress σ (simulation units). The displacement field (colorbar) within the cone is approximately radially uniform. **c.** A series of cusped profiles $Z(R)$ for progressively increasing $|\sigma|$. Each profile is inverted (see inset) and vertically shifted by Z_0 such that the cusp tips align. The power law $Z - Z_0 \sim R^{1/4}$ for the tip profile, as predicted in Eq. (S5), is overlaid for two example data sets.

To demonstrate the cone-cusp transition predicted above, the simple ball-spring mesh of Fig. 3 does not capture the continuum behaviour of the cone at its tip, unless an excessively fine mesh is used. Instead, here we use a finite-element elasticity simulation implemented in COMSOL. Figure S1**a** shows the initial geometry: we construct a cone of tip angle $\theta = 20°$ and height L (simulation units), composed of an approximately incompressible material of Young's Modulus $E = 10^6$ (simulation units) and Poisson ratio $\nu = 0.49$. The cone is anchored at its base, with the tip free to move. To implement the bending rigidity necessary for continuum active elastocapillarity (as discussed in the main text and Section C) we coat this cone in a thin layer of stiffer material, $E = 10^9$, $\nu = 0.49$, with a thickness $d = 10^{-4}L$. The negative surface tension is implemented by imposing an initial dilational stress σ throughout this thin layer.

Figure S1**b** shows an example of the resulting shape, for $\sigma = -10^{13}$ (simulation units). The cone dramatically elongates and sharpens, adopting a cusped profile. The resulting displacement field (Fig. S1**b**, colorbar) is indeed approximately uniform across the width of the cone, an assumption made in the derivation above. Figure S1**c** overlays a series of cusp profiles $Z(R)$ for increasing $|\sigma|$. Each profile is inverted (i.e., Z increases away from the cusp tip, see inset) and shifted vertically such that the cusp tips align. Shown also are two example best-fit $R^{1/4}$ power laws, as predicted by Eq. (S5) above. We see that, as $|\sigma|$ increases, the cone tip progressively elongates, and its sides are drawn in. Further, as $|\sigma|$ increases, we find a regime in which the displacement field within the cone is approximately radially uniform (Fig. S1**b**) and an $R^{1/4}$ power law provides a good fit for the profile, although unsurprisingly with some deviation at the fixed cone base and singular tip.

B. Modifications to active elastocapillarity: the effect of viscosity and bulk dispersion

1. The effect of viscosity

In the main text, we have focused on inertial dynamics, but we may easily consider the effects of viscosity using a Kelvin-Voigt viscoelastic shear modulus,

$$\mu(\omega) = \mu - i\nu\omega, \tag{S6}$$

in Eq. (20). For simplicity, we focus on the overdamped limit, neglecting inertia. In this limit, Eq. (20) simplifies to

$$i\nu\omega - \mu - \frac{\gamma}{2}q - \frac{\kappa}{2}q^3 = 0, \tag{S7}$$

or in dimensionless units

$$i\tilde{\omega}_\nu - 1 + \frac{1}{2}\tilde{q}_\gamma - \frac{1}{2}\tilde{\kappa}_\gamma\tilde{q}_\gamma^3 = 0, \tag{S8}$$

where $\tilde{\omega}_\nu = \nu\omega/\mu$. Unlike some other examples of active solids [14], here there is no phase lag between driving and response, so $\tilde{\omega}_\nu$ is always purely imaginary and we do not see overdamped waves. However, our tuneable shape instability remains, with the critical wavenumber and bending modulus as in Eq. (25).

2. *Bulk dispersion*

In the main text, we focused on a bending modulus as the regularizing mechanism for the $q \to \infty$ limit of active elastocapillarity. The lengthscale that κ introduces, l_κ, comes from the surface physics. An alternate additional lengthscale, l_μ, comes instead from stabilising higher-order gradients in the the bulk physics. In Fourier space, we consider a q-dependent 3D shear modulus

$$\mu(q) = \mu_0 + \mu_1 q^2 + ..., \tag{S9}$$

where $l_\mu = \sqrt{\mu_1/\mu_0}$ and $\mu_1 > 0$. We can assess the effects of such higher-order terms using Eq. (20), by setting $\kappa = 0$ and sending $\mu \to \mu + \mu_1 q^2$. The result is a new dispersion,

$$\rho\omega^2 - \frac{4\mu(q)q^2\alpha_T}{q + \alpha_T} - \gamma q^3 = 0, \tag{S10}$$

where now $\alpha_T = \sqrt{q^2 - \rho\omega^2/\mu(q)}$. In the absence of surface tension, Eq. (S10) admits Rayleigh waves $\omega \sim \sqrt{\mu(q)/\rho q} \sim \mu_1 q^2)$ as $q \to \infty$. By contrast, pure capillary waves scale as $\omega \sim \gamma q^{\frac{3}{2}}$. A power count thus indicates that bulk dispersive effects also regularize the large q limit. The asymptotic behaviour of Eq. (S10) as $q \to \infty$ is

$$\rho\omega^2 + |\gamma|q^3 - \xi^2\mu_1 q^4 = 0, \tag{S11}$$

where $\xi = 0.955...$ is the ratio of Rayleigh to bulk wave velocity [34]. In dimensionless form, Eq. (S11) is

$$\tilde{\omega}_\gamma^2 + \tilde{q}_\gamma^3 - \xi^2\tilde{\mu}_\gamma\tilde{q}_\gamma^4 = 0. \tag{S12}$$

where $\tilde{\mu}_\gamma = (l_\mu/l_\gamma)^2$. Comparing Eq. (S11) to Eq. (20), we see the effects of bulk dispersion on high wavenumbers are qualitatively similar to a bending modulus, but the exact scalings differ. High wavenumbers are stabilised as q^5 with the bending modulus κ, but as q^4 with bulk dispersion μ_1. Instead of $\tilde{\kappa}_\gamma = (l_\gamma/l_\gamma)^3$ controlling the phase planes Fig. 2**d**–**e**, we have $\tilde{\mu}_\gamma = (l_\mu/l_\gamma)^2$. These dimensionless variables scale differently with their associated lengthscales l_κ and l_μ. One consequence of this difference would be a shifted scaling of the phase boundaries in Figs. 2**d**–**e**.

C. Active elastocapillarity and surface wrinkling

The wrinkling of a surface is an example of dilational surface stresses leading to 3D shape change in an elastic solid. Wrinkles are generated by mechanisms such as bonding a thin pre-stressed sheet onto a flat elastic layer [50], the differential swelling of an oxidised surface on a compliant substrate [27, 42, 47], the depressurization of a thick elastic sphere coated with a stiff film [48, 49], or biological growth [42, 44, 45]. Ref. [48] derives a surface energy modelling the formation of wrinkles, which contains an effectively negative surface tension regularized by higher-order gradients (bending rigidity) and nonlinear terms in the normal surface displacement.

Here we discuss the distinctions between our results and those found in the wrinkling literature, and demonstrate that we recover classical wrinkling results in the limit where the wavelength of instability q^{-1} is much smaller than the sphere radius R, c.f. Figs. 2**d**–**f** in the main text. We focus on two key distinctions. First, at the level of linear analysis, we treat instabilities with wavelengths comparable to the system size, whereas the treatment of wrinkles assumes wavelengths small compared to system size. Second, at the level of nonlinear analysis, the wrinkling literature focuses on the role of surface curvature as a symmetry breaking parameter, which selects one wrinkling pattern over another [47]. Our treatment of nonlinear deformations is focused on the selection of global shape, where material nonlinearity breaks the symmetry between positive and negative strain ϵ, c.f. Eq. (3) in the main text.

To couple bulk elastic response to a surface energy, two approaches are possible. In the first, one models bulk elasticity with an elastic foundation ('Winkler mattress') model [61], in which displacement u is assumed to be

proportional to the locally applied pressure, with no shear coupling between adjacent material elements. This approximation amounts to adding an an external potential to a surface-only free energy. To quadratic order, the combined surface-bulk potential reads

$$F = \int d^2x \left[\frac{K}{h} u^2 + \frac{\gamma}{2} \left(\nabla u\right)^2 + \frac{\kappa}{2} \left(\nabla^2 u\right)^2 \right]. \tag{S13}$$

In Eq. (S13) the term $(K/h)u^2$ is the external potential, with K the Winkler modulus [62, 63] of the foundation, and h the foundation thickness. This is the approach taken in the wrinkling literature [46–49]. Equation (S13) has also been proposed to model dynamical phenomena such as cytoskeletal wave propagation and membrane-cytoskeleton fluctuations [60]. The approach Eq. (S13) has the advantage of being able to transparently model nonlinear bulk response by adding higher-order potential terms: $V_1u^3 + V_2u^4 + \cdots$ [48]. Further, by minimizing a single functional Eq. (S13), one derives a nonlinear PDE (for example, of Swift-Hohenberg type [48]) governing the selection of dimples or labyrinthine patterns [46, 48, 49]. The disadvantage is that Eq. (S13) is not translationally invariant (i.e. invariant under $u \to u+a$). Thus, while Eq. (S13) is appropriate for modelling nonlinear pattern selection when the wavelength of instability is small compared to the system size, this approach cannot be applied when studying global deformations.

A distinct approach, taken throughout our work, is to model bulk elasticity using the fully 3D free energy [34]. To quadratic order, one has

$$F = \int d^3x \left[B u_{ii}^2 + \mu \left(u_{ik} - \delta_{ik} u_{ll}\right)^2 \right] + \int d^2x \left[\frac{\gamma}{2} \left(\nabla u\right)^2 + \frac{\kappa}{2} \left(\nabla^2 u\right)^2 \right]. \tag{S14}$$

Equation (S14) is translationally invariant (invariant under both $u_i \to u_i + a,\ u \to u + a$), and can be used to model linear instabilities of wavelength comparable to the system size, as is done in Fig. 2 of the main text. However, in contrast to Eq. (S13), variation of Eq. (S14) leads to two sets of equations: the first encoding the bulk elasticity problem and the second encoding the stress-matching boundary condition. Solving both gives the dispersion relations found in the Methods.

A comparison of these approaches amounts to a discussion of the validity of the elastic foundation model [62, 63]. The dispersion Eq. (20) in the Methods maps to the dispersion of Eq. (S13) (see also the dispersion of Ref. [48]) under the identification $\mu q = K/h$, i.e. the foundation thickness (or Winkler modulus K) scales with the perturbation wavelength. This same scaling occurs when matching Hertzian contact mechanics to contact on an elastic foundation [61].

To complete the mapping between dispersions, we must identify our bending modulus κ and negative surface tension γ with the material parameters of a stiff layer bonded onto the elastic substrate. We identify the bending stiffness κ with that of a thin layer of thickness h and elastic modulus μ_f, $\kappa \sim \mu_f h^3$ [34]. Equivalently, the bendoelastic length scales with the film thickness, $l_\kappa \sim (\mu_f/\mu_s)^{1/3} h$. Our negative surface tension γ (a force per unit length) maps to a dilational stress σ (a force per unit area), $|\gamma| \sim \sigma h$. With these mappings, our predictions for linear instability coincide with those of Ref. [48] and classical results [46, 47]. In particular, in the Methods we derive a critical wavenumber $q^* \sim (\kappa/\mu)^{-1/3}$ for instability, with threshold negative surface tension $|\gamma^*| \sim \kappa^{1/3}\mu^{2/3}$. With the mappings above, this threshold reads

$$\sigma^* \sim \mu_f \left(\frac{\mu}{\mu_f}\right)^{2/3}, \tag{S15}$$

$$q^* \sim \frac{1}{h} \left(\frac{\mu}{\mu_f}\right)^{1/3}, \tag{S16}$$

in agreement with classical results, for example Ref. [46] Eqs. (3.4), (3.5). However, we emphasize that our focus is on the opposite limit, $q^{-1} \sim R$, in which translational invariance cannot be neglected, and these classical results do not apply.

Pursuing the analysis Eq. (S14) at the nonlinear level will select modes, analogously to the selection of dimpled or labyrinthine patterns observed in wrinkling [27, 42, 46, 47] and rationalized via (for example) a modified Swift-Hohenberg equation [48]. However, rather than work with the full nonlinear equations directly, we focus on the case of global shape change and assume homogeneous deformations from the outset. The nonlinear mechanical energy functional then simplifies to a function of a single variable, the stretch factor λ, c.f. Eq. (2). This simplification highlights the role of material nonlinearity as a novel symmetry breaking mechanism, distinct from the role of surface curvature in wrinkling [46–48]. In Eq. (3) of the main text, we demonstrate that material nonlinearity can break the symmetry between elongated worm-like shapes and flattened pancakes, leading to the phase diagrams shown in Fig. 3.

D. Degenerate material nonlinearity: the Gent model

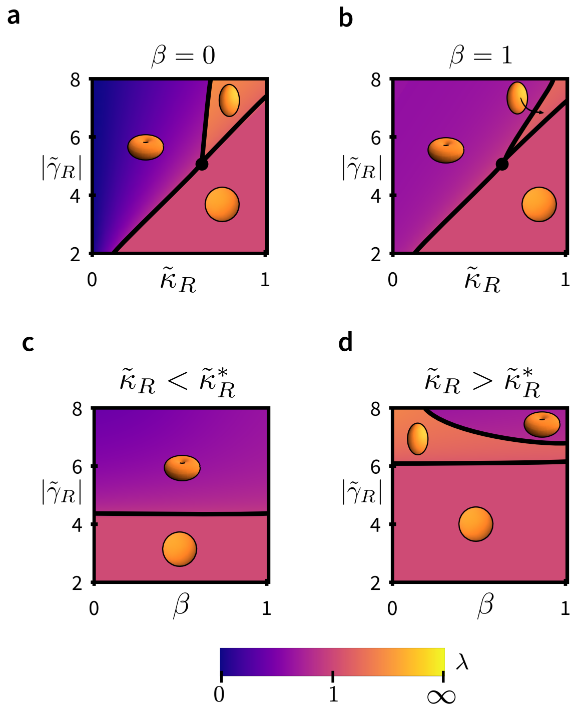

FIG. S2: **Phase diagram of the Gent model**. **a–b.** Cuts in $\tilde{\kappa}_R$–$|\tilde{\gamma}_R|$ space for the neo-Hookean limit $\beta = 0$ (**a**), and a generic nonzero β (**b**). The nonlinearity β does not change the location of the critical point. **c–d.** Cuts in β–$|\tilde{\gamma}_R|$ space above and below $\tilde{\kappa}_R^*$, showing a wormlike region opening up without a critical point.

In the main text, we explored the effects of material nonlinearity on the phase diagram of an active elastocapillary sphere, using the Mooney-Rivlin model as a minimal example. The Landau expansion for Mooney-Rivlin, Eq. (37), has the nonlinearity parameter $\tilde{\alpha}$ entering at cubic order in ϵ, as is generically expected. The structure of the phase diagram Fig. 3**b** stems directly from this cubic term, and as such should be preserved across different choices of strain energy. Here, we investigate how this structure changes in the degenerate case in which nonlinearity only enters at quartic (or higher) order. A common nonlinear elastic model which exhibits this degeneracy is the Gent model [66]:

$$f_{\text{Gent}} = -\frac{\mu}{2\beta} \log\left(1 - \beta(I_1 - 3)\right). \tag{S17}$$

Gent elasticity is a correction to neo-Hookean behaviour at high extensions, used to model rubbers [66] and biological tissues [67]. The material nonlinearity parameter β models finite chain extensibility, with divergences in f_{Gent} occurring as $\lambda \to 0$ ($\lambda \sim \beta$) or $\lambda \to \infty$ ($\lambda \sim 1/\sqrt{\beta}$). Taking $\beta \to 0$ gives neo-Hookean elasticity.

Expanding Eq. (S17) as we did for the Mooney-Rivlin model Eq. (35) we obtain

$$f_{\text{Gent}} = \frac{3\mu\epsilon^2}{2} - \mu\epsilon^3 + \frac{1}{4}(9\beta + 4)\mu\epsilon^4 + ..., \tag{S18}$$

in which β enters at quartic order. The result is that the Gent model behaves essentially as a neo-Hookean solid for all β.

Equation (36) gives the location of the critical point within the Mooney-Rivlin model. It describes a line in $\tilde{\alpha}$–$\tilde{\kappa}_R$–$|\tilde{\gamma}_R|$ space. This line intersects a generic coordinate plane to exhibit the critical point. In the Gent model, this line is parallel to the β axis, and so a cut in material nonlinearity space will not exhibit a critical point. However, a generic cut, including the $\tilde{\kappa}_R$–$|\tilde{\gamma}_R|$ plane, will. In Fig. S2**a–b**, we show the $\tilde{\kappa}_R$–$|\tilde{\gamma}_R|$ plane of the Gent phase diagram, for $\beta = 0$ (the neo-Hookean limit) and a generic nonzero β. The phase plane contains a critical point, but upon varying β its location does not change, as is generically expected. Rather, the wormlike region of the phase plane simply narrows. The location of the critical point can be found by setting $\tilde{\alpha} = 0$ in Eq. (36), giving $\tilde{\kappa}_R^* = 5/8$. In Figs. S2**c–d** we show the β–$|\tilde{\gamma}_R|$ plane below and above $\tilde{\kappa}_R^*$. The locus of the critical point runs parallel to these cuts, and we do

not see a critical point in these diagrams. Instead, at low bending modulus, pancakes are favoured for all β. At high bending modulus, a wormlike region opens up, separated from pancakes by a curve running to $\beta \to \infty$.

E. Supplementary Movie

The Supplementary Movie shows animations of the particle-based simulation results given in Figs. 3**d**–**e** of the main text. The movie shows an active elastocapillary sphere destabilising as active driving $|\tilde{\gamma}_R|$ is increased. The sphere destabilises to a pancake for small $\tilde{\alpha}$ or a worm for large $\tilde{\alpha}$, and exhibits a snap-through transition at intermediate $\tilde{\alpha}$.

---

* Electronic address: a.souslov@bath.ac.uk
